# Oesophageal Perforation Surgical Treatment: What Affects the Outcome? A Multicenter Experience

**DOI:** 10.3390/jcm14124019

**Published:** 2025-06-06

**Authors:** Antonio Giulio Napolitano, Dania Nachira, Leonardo Petracca Ciavarella, Eleonora Coviello, Domenico Pourmolkara, Rita Vaz Sousa, Elisa Meacci, Tiziano De Giacomo, Federico Venuta, Venanzio Porziella, Stefano Margaritora, Francesco Puma, Jacopo Vannucci

**Affiliations:** 1 Department of General Thoracic Surgery, Fondazione Policlinico Universitario “A. Gemelli”, IRCCS, Università Cattolica del Sacro Cuore, 00136 Rome, Italy; antoniogiulionapolitano@gmail.com (A.G.N.); dania.nachira@policlinicogemelli.it (D.N.); leonardo.petraccaciavarella@policlinicogemelli.it (L.P.C.); elisa.meacci@policlinicogemelli.it (E.M.); venanzioporziella@gmail.com (V.P.); stefano.margaritora@policlinicogemelli.it (S.M.); 2 Department of Thoracic Surgery, University of Perugia Medical School, Ospedale Santa Maria della Misericordia, 06129 Perugia, Italy; domenico.pourmolkara@gmail.com (D.P.); francesco.puma@unipg.it (F.P.); jacopovannucci@tiscali.it (J.V.); 3 Division of Thoracic Surgery and Lung Transplant, AOU Policlinico Umberto I, University of Rome Sapienza, Viale del Policlinico 155, 00161 Rome, Italytiziano.degiacomo@uniroma1.it (T.D.G.); federico.venuta@uniroma1.it (F.V.)

**Keywords:** oesophageal perforation, mediastinitis, oesophagectomy, surgical emergency

## Abstract

**Background:** Oesophageal perforation (OP) is a life-threatening condition requiring prompt diagnosis and treatment. Mortality is influenced by several factors, such as aetiology, defect location, comorbidities, age, and delays in treatment. This study reviews patients with OP undergoing surgery, analysing mortality risks and the impact of timing on surgical outcomes. **Methods:** Medical records of 45 patients surgically treated for OP across three tertiary centers were analysed. **Results:** Of the 45 patients, 31 were male (68.88%) and 14 were female (31.11%), with a mean age of 66.00 ± 17.75 years. Pre-operative CT was performed in all patients, and 18 (40%) underwent oesophagogastroduodenoscopy. As many as 25 patients (55.55%) presented within 24 h, 10 (22.22%) within 24–72 h, and 10 (22.22%) after 72 h. Symptoms included pain, vomiting, fever, dysphagia, and subcutaneous emphysema. Foreign body ingestion and Boerhaave’s syndrome were the leading causes (33.33% each), followed by caustic ingestion (17.77%) and iatrogenic and traumatic cases. Treatments included primary repair, debridement, oesophagectomy, and oesophagogastrectomy. Primary repair was performed in 22 cases (48.88%), and muscle flaps reinforced 11 of these. Direct repair showed the highest success rate when performed within 24 h. Thirty patients (66.66%) experienced complications, including respiratory failure, oesophagopleural fistula, and sub-stenosis. The hospital stay average was 36.34 ± 35.03 days. Nine patients underwent same-session/two-stage gastroplasty or retrosternal coloplasty for reconstruction, with complications including stenosis and leaks. Six patients (13.33%) died within the first 24 h after surgery, primarily due to severe comorbidities (three (50%) were octogenarians). **Conclusions:** OP is a life-threatening condition with high mortality. Primary repair is the preferred treatment. Oesophagectomy and gastrectomy are reserved for extensive lesions. Muscle flaps can reinforce sutures in cervical and thoracic perforations. Mortality is mainly influenced by the severity of the patient’s clinical picture and comorbidities, rather than by time and type of treatment.

## 1. Introduction

Oesophageal perforation (OP) is a potentially life-threatening clinical condition that requires prompt diagnosis and timely intervention. Although oesophageal perforations are relatively rare, several retrospective analyses have been conducted over the years to establish appropriate diagnostic and treatment pathways. In 2018, the World Society of Emergency Surgery (WSES) published guidelines that have become a benchmark in this field [1].

The mortality associated with OP is influenced by several factors, including aetiology, the location of the perforation along the oesophageal tract, patient comorbidities, age, and the interval between symptoms onset and diagnosis. When addressed in an emergency setting (within 24 h), the mortality rate ranges from 10% to 25%. However, if treatment is delayed beyond 48 h, mortality can reach up to 60% [2,3]. Recently, outcomes have improved, likely due to advancements in diagnostic and therapeutic techniques, the adoption of a multidisciplinary approach, and improvements in medical support systems [4].

Spontaneous and iatrogenic perforations show a higher mortality rate compared to those caused by retained foreign bodies. According to Sdralis et al., thoracic perforations (accounting for 73% of cases) and abdominal perforations are associated with higher mortality rates than cervical perforations [5]. The clinical presentations of OP vary widely and may include mediastinitis, pneumomediastinum, pneumothorax, pleural empyema, peritonitis, and, in severe cases, progression to sepsis and multiorgan failure [4]. The lower risk associated with cervical perforations is attributed to the anatomical environment, which limits rapid contamination and the spread of infection [6].

This study aims to analyse the outcomes of patients undergoing emergency or urgent surgery for OP and to evaluate the factors influencing survival in managing this condition.

## 2. Methods

### 2.1. Study Design

Among 170 cases of OP recorded in the participating institutions, we retrospectively analysed 45 patients who required emergency or urgent surgical operation between January 2014 and December 2023. These cases involved perforations with different aetiologies, all characterised by complete tearing, which precluded endoscopic treatment as a viable option.

The exclusion criteria were as follows:

- Oesophageal perforations, ischaemia, and fistula occurring after elective oesophageal surgery.
- Patients treated exclusively with endoscopic approaches.
- Oesophageal tumours causing perforations.

### 2.2. Diagnosis and Symptoms

The diagnosis of OP was established using a combination of instrumental investigations, including computed tomography (CT), endoscopy, chest X-ray, and/or oesophagogram with swallowed contrast, along with clinical examination.

Symptoms varied based on the timing and anatomical location of the oesophageal rupture [7]. The most reported initial symptoms included acute chest pain (sometimes radiating to the neck or shoulders), dyspnoea, fever, vomiting, and subcutaneous emphysema [8]. For cervical perforations, specific symptoms such as sialorrhea, dysphagia and odynophagia were particularly frequent [9].

Contrast oesophagography using water-soluble oral contrast was effective in identifying leaks, while chest X-rays, although less specific, revealed indirect signs of perforation such as pneumothorax, subcutaneous emphysema, and pleural effusion [10]. In most cases, patients presented with a suggestive clinical picture, leading to an initial diagnostic CT scan.

**Figure 1:**
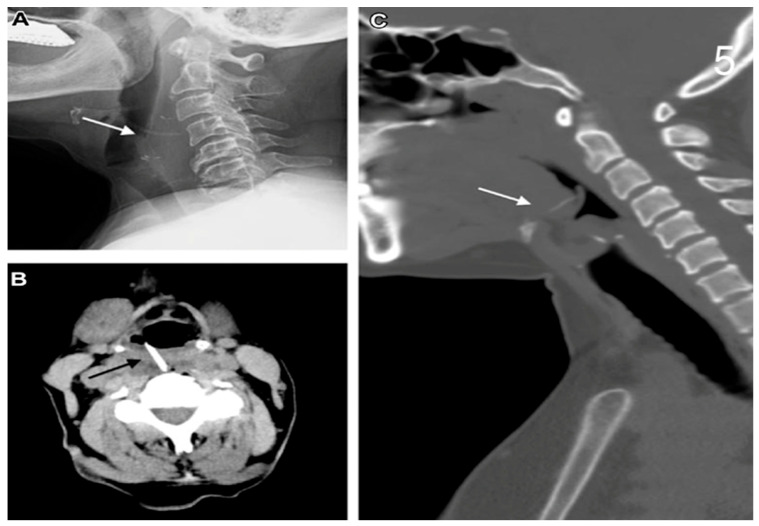
(**A**) Cervical X-Ray showing FB (chicken bone). (**B**) Cervical CT scan showing FB, hyperdense body. (**C**) CT cervical scan showing rectilinear body.

**Figure 2:**
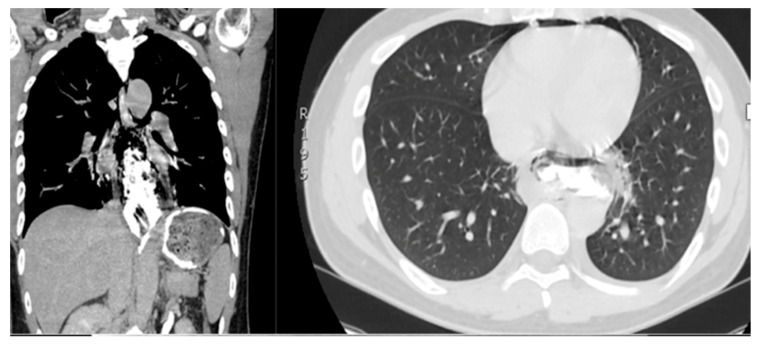
CT scan showing oesophageal perforation after ingestion of FB.

**Figure 3:**
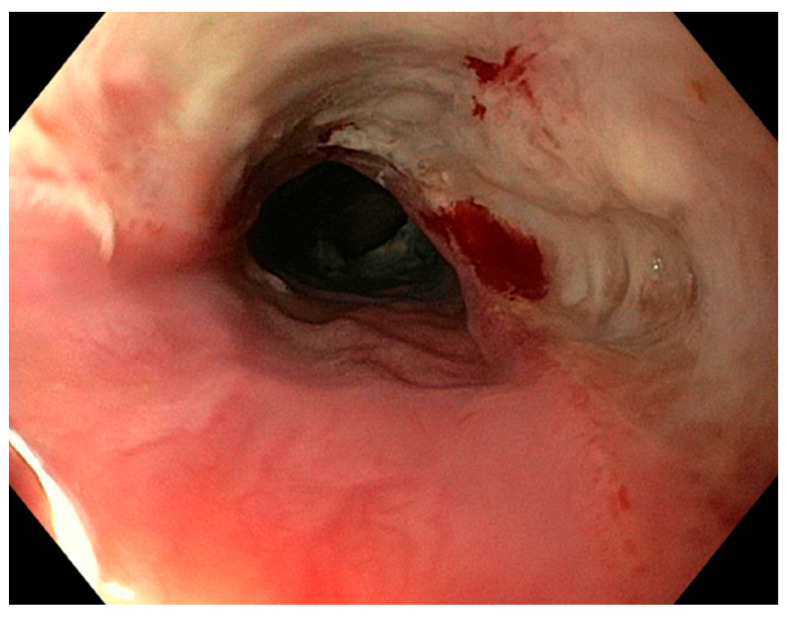
EGDS, showing oesophagus after caustic ingestion of a battery.

The CT scan is extensively used in clinical practice due to its reliability in confirming the diagnosis and in accurately locating the perforation site (Figure 1A–C and Figure 2) [11]. When feasible, endoscopy was employed to assess the size of the perforation and to evaluate the presence of oesophageal necrosis (Figure 3). Although the role of endoscopy in cases of OP is debated due to the potential risk of further damage, it was performed in selected cases with diagnostic benefits [12].

Rigid oesophagoscopy (RE) has proven to be useful in specific scenarios, particularly as a preliminary step for surgery to remove bulky retained foreign bodies prior to definitive surgical repair [13].

### 2.3. Management

Surgery was performed as an emergency within 24 h, an urgent intervention within 24–72 h, or after 72 h in cases of delayed diagnosis or late referral to the emergency department. The surgical approach was tailored to the primary cause, the location of the perforation (cervical, distal, oesophagogastric), the extent of the lesion, the timing, and the condition of the surrounding tissues.

Surgical management included primary repair of the perforation, debridement, drainage of the pleural cavity and/or cervical area, and, in severe cases, oesophageal resection with or without gastrectomy. These procedures were often combined with oesophagostomy and jejunostomy to ensure nutritional support.

**Figure 4:**
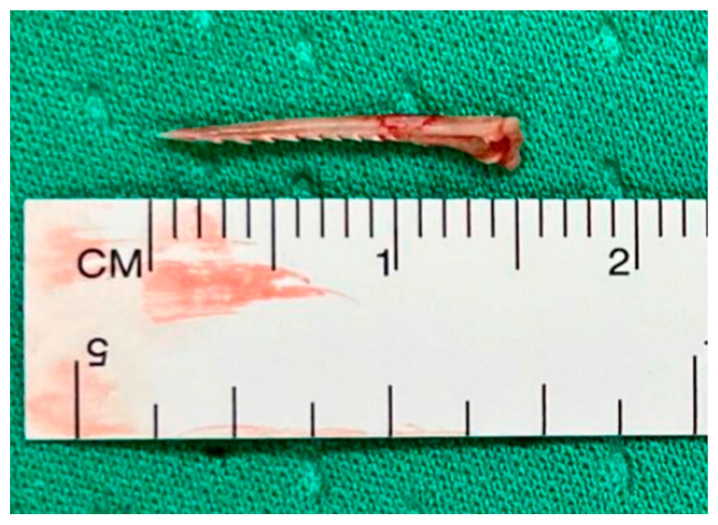
Foreign body removal after direct repair.

**Figure 5:**
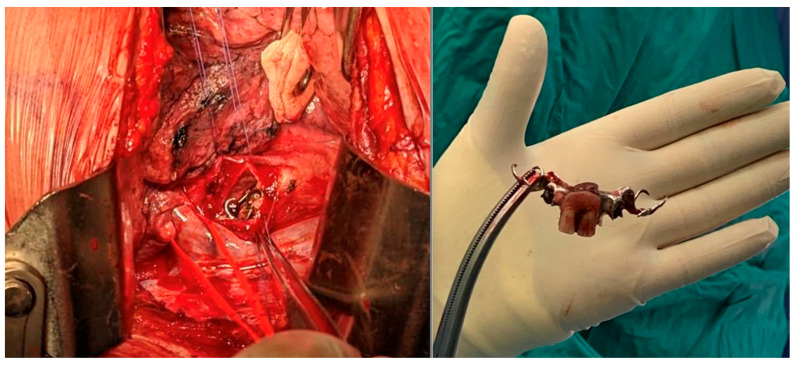
Oesophageal perforation for FB ingestion—dental prosthesis removed from oesophagus.

**Figure 6:**
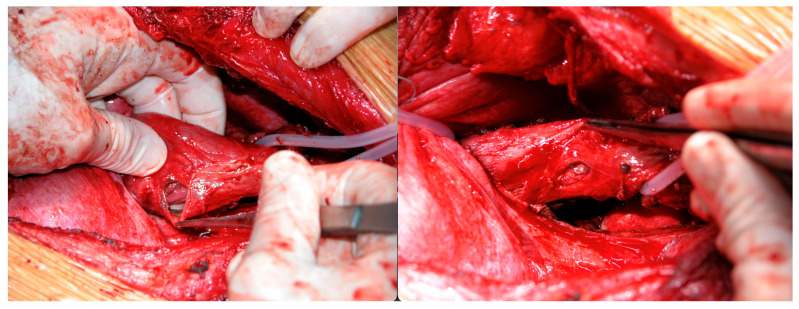
Isolation of the oesophagus and identification of oesophageal rupture.

**Figure 7:**
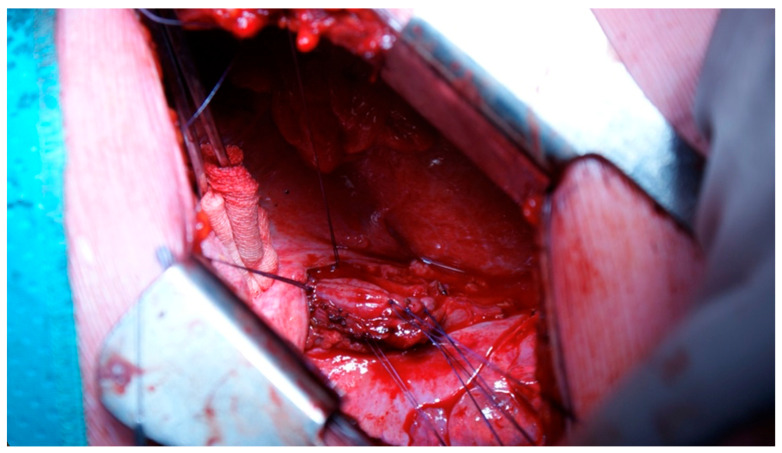
Direct repair of oesophageal rupture with Vicryl 4/0 and muscular plane with Vicryl 3/0.

For OP caused by foreign bodies, trauma, iatrogenic injuries, or Boerhaave’s syndrome within 24 h, oesophagotomy with foreign body removal (Figure 4) and direct repair (<2 cm without necrosis) was performed via thoracotomy or cervicotomy (Figure 5) [14]. After isolating the oesophagus (Figure 6) and removing necrotic tissue, the mucosal plane was sutured using 4/0 Vicryl interrupted stitches, and the muscular plane was sutured with 3/0 Vicryl sutures (Figure 7) [15].

**Figure 8:**
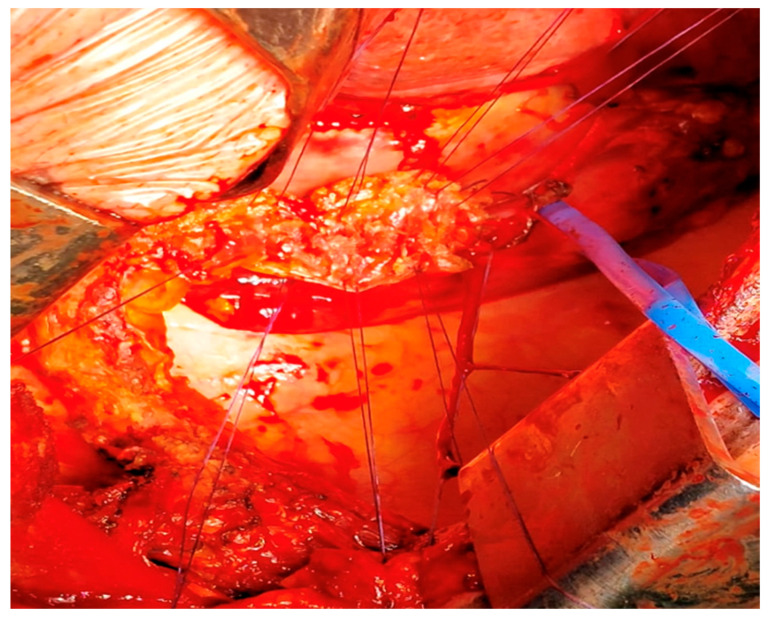
Pedicle flap prepared to be placed on oesophageal thoracic rupture as reinforcement.

A pedicled flap was used to reinforce the suture site, with options based on perforation location, including intercostal muscle, cervical muscle, pleural, omental, or diaphragmatic tissue flaps (Figure 8) [16,17,18,19].

**Figure 9:**
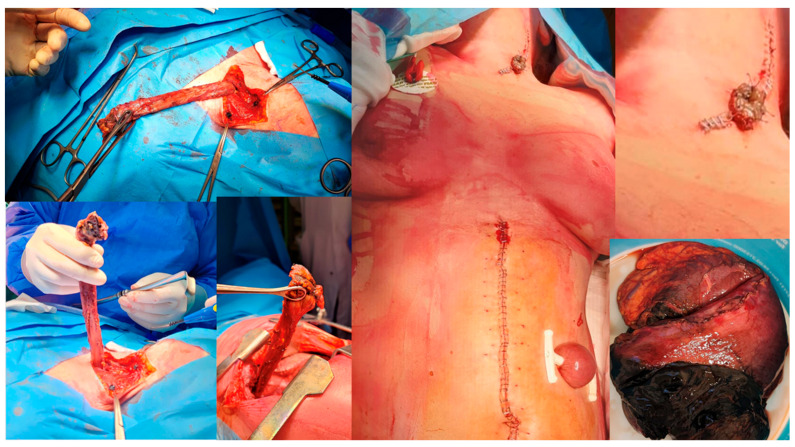
Oesophagogastrectomy after caustic ingestion.

For cases presenting after 72 h with cervical abscesses, pleural empyema, or an unclear perforation site, debridement and drain placement were the primary treatment [20]. Patients with Boerhaave’s syndrome or caustic ingestion involving the distal oesophagus and/or gastric perforations underwent oesophagectomy or oesophagogastrectomy with cervical oesophagostomy (Figure 9) [21,22,23].

In patients with septic shock or systemic infection beyond 72 h, oesophageal diversion [24] was combined with gastrostomy or jejunostomy to initiate feeding support. Jejunostomy was routinely placed in all major oesophageal resections for long-term nutritional support [25].

**Figure 10:**
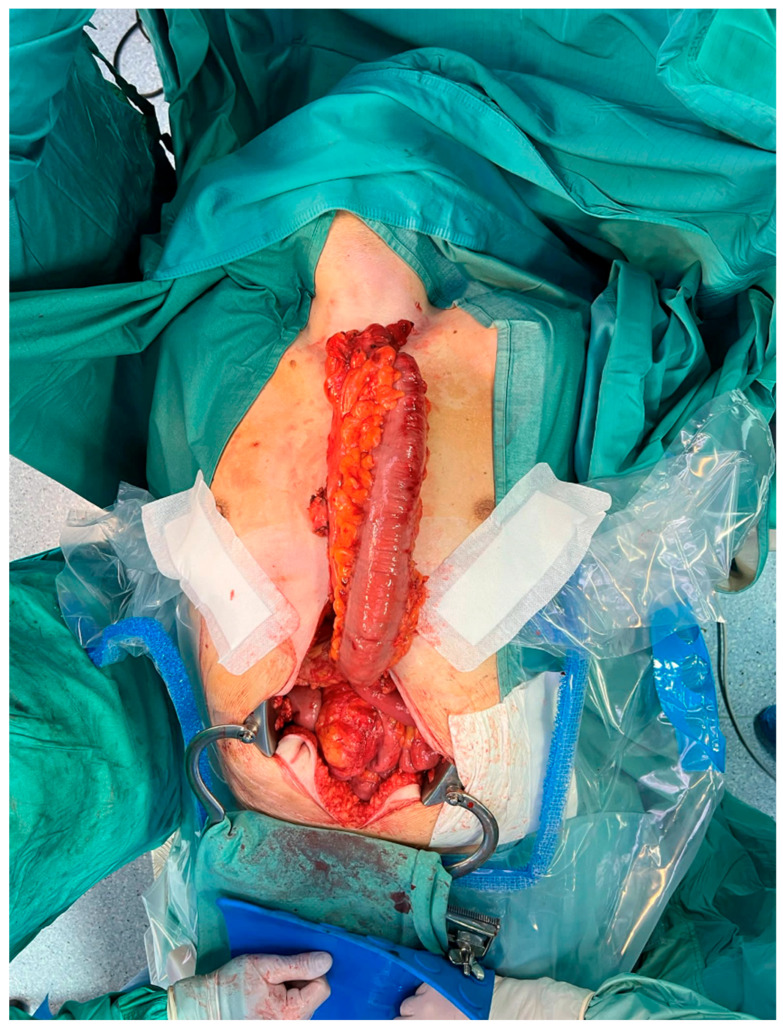
Coloplasty second stage recanalization surgery.

Reconstruction with gastroplasty during the primary operation was rarely performed and only in specific cases, such as those without caustic ingestion or significant cervical/pleural infections. Otherwise, infection control and stabilisation were prioritised, with reconstruction delayed to a second phase following endoscopic evaluation (EGDS) to exclude stenosis or scars. Selected patients underwent retrosternal oesophagocoloplasty [26] or oesophagogastric bypass [27] for reconstruction (Figure 10).

All patients received perioperative broad-spectrum antibiotic therapy. Postoperatively, most patients were admitted to the ICU for haemodynamic support and monitoring for at least 24 h.

### 2.4. Statistical Analysis

Continuous variables were expressed as mean and standard deviation, categorical variables as mean and percentage (%). Student’s *t*-test was used to compare means between two continuous variables. Categorical variables were compared by a χ^2^ test. A *p*-value < 0.05 was considered statistically significant. Statistical analysis was performed using IBM SPSS Statistics for Macintosh, Version 25.00 (Armonk, NY, USA).

## 3. Results

A total of 45 patients were evaluated: 31 male (68.88%) and 14 female (31.12%), with a median age of 66.00 ± 17.75.

Within 24 h of the event, 25 (55.55%) patients were treated following early-onset symptoms, including 3 out of 4 who were as iatrogenic and treated in the same operative session.

Moreover, 10 patients were referred within 24–72 h and 10 after 72 h. The main clinical baseline characteristics and symptoms of patients are reported in Table 1.

The most common symptoms included chest pain in 26 cases (57.77%), vomiting in 12, fever in 10, abdominal pain in 8, subcutaneous emphysema in 5, and dysphagia in 5. Boerhaave’s syndrome was diagnosed in 10 out of 15 patients (66.66%), either with vomiting alone or vomit associated with chest/abdominal pain, melena, dysphagia, and sialorrhea.

**Figure 11:**
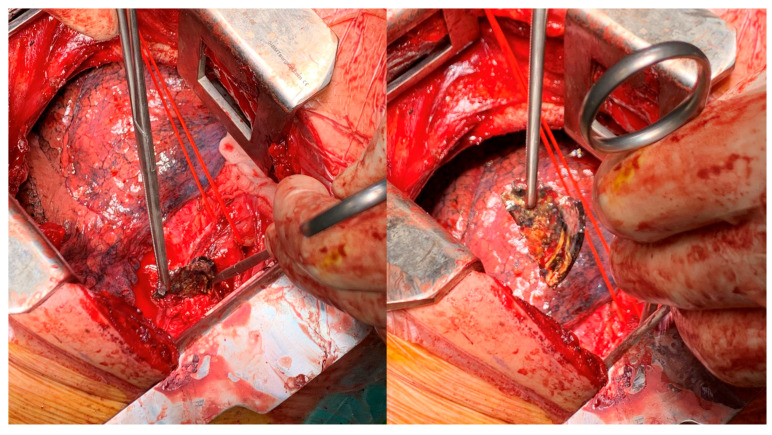
Removal foreign body in thoracotomy.

The surgical approach, via cervicotomy, thoracotomy (Figure 11), or laparotomy, was chosen depending on the different OP localisation.

Table 2 summarises the causes, timing, location, and surgical procedures.

OP treatment was performed through direct repair in 22 patients (48.88%); in 11 cases (50%), suture was reinforced with flaps (intercostal, cervical, diaphragm muscle, or mediastinal tissues). Direct suturing was employed in patients presenting within 72 h of the leak.

**Figure 12:**
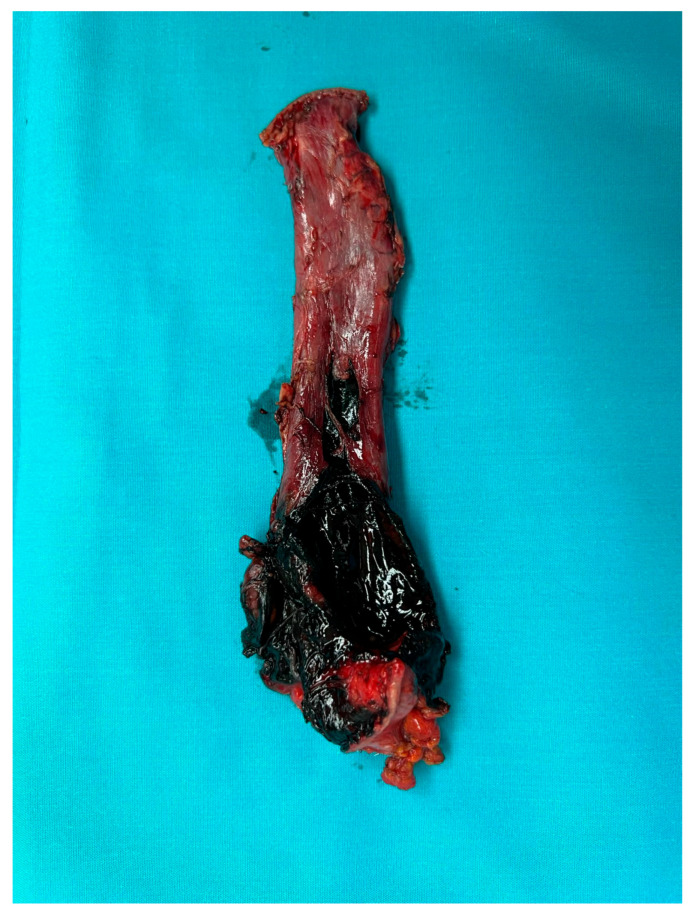
Oesophagectomy after caustic ingestion.

Oesophageal resection, oesophago/gastric resection, and bipolar diversion were performed in 12 patients (26.66%); all these patients were affected by Boerhaave’s syndrome and caustic ingestion (Figure 12).

**Figure 13:**
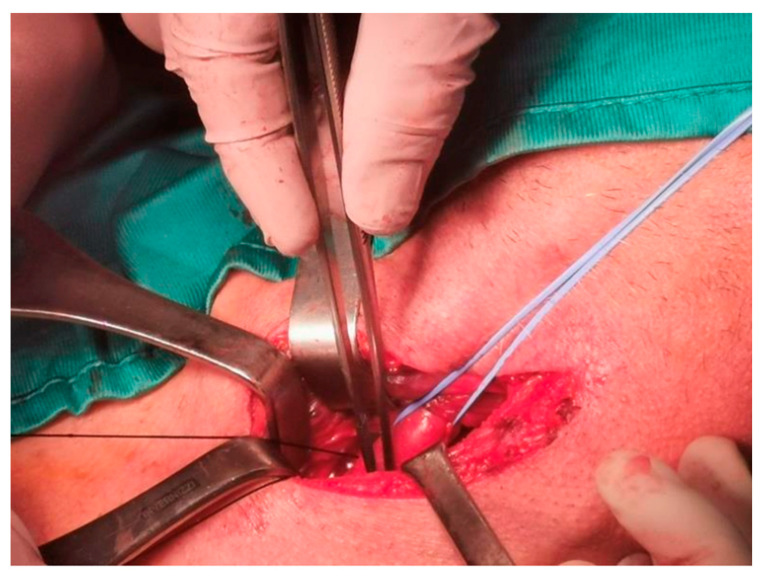
Cervical oesophageal preparation for bipolar exclusion.

Two cases of Boerhaave’s syndrome underwent secondary oesophageal bipolar diversion (Figure 13) due to a septic clinical condition and general worsening after first direct surgical repair.

Nine patients had a final digestive reconstruction—two patients in the same operative session after oesophagectomy through gastroplasty, while in seven patients, the reconstruction was performed in two stages through retrosternal coloplasty. In five of these patients, reconstruction was performed after 5.20 ± 1.32 months, and retrosternal gastroplasty was carried out in the remaining patients (28.57%) after 19.50 ± 0.5 days.

Nutritional jejunostomy was necessary in all patients undergoing oesophageal and/or gastric resection due to the length of recovery without oral feeding and poor clinical conditions.

In 15 patients, the clinical course was regular, and 12 (80%) of these patients were treated with direct suture repair within 24 h of the occurrence of OP. There were no statistically significant differences in the group of patients with muscle flap positioning (cervical vs. thoracic) in terms of post-operative complications (*p*: 0.303) and mortality (*p* = 0.428) (Table 3).

The complications observed in the remaining 30 patients are listed in Table 3, and the most frequently recorded were fever (17 patients), respiratory failure (6 patients), and oesophagopleural fistula (7 patients).

After two-stage reconstructive surgery, the most common complications were stenosis in two cases, treated with endoscopic dilatation, fever in two patients, and leak in two patients treated by cervicotomy and packing.

The average hospital stay was 36.34 ± 35.03 days. The longest recovery was observed in two patients with Boerhaave’s syndrome and major post-operative complications. Moreover, the mean hospitalisation time was 34.33 ± 16.86 days for patients undergoing retrosternal delayed reconstruction. The average intensive care unit stay was 6.58 ± 4.06 and was necessary for 28 out of 45 patients.

The mortality rate was 13.33% (six cases).

In patients with a negative swallow test, oral nutrition was initially restored with a semi-liquid diet, progressing to soft food after 20.07 ± 14.05 days.

For patients with suicidal intent, psychiatric treatment was initiated. All patients at 3-month follow-up showed complete return to oral feeding, despite the patients developing stenosis, later dilated. Despite delayed reconstruction in some patients, all patients resumed oral feeding by the 3-month follow-up, either after reconstruction or with temporary nutritional support via jejunostomy. Of these, eight patients received bipolar exclusion as a primary strategy due to extent of necrosis or contamination, and four underwent it after failure of initial repair. Three patients died, all of whom had severe sepsis and multi-organ dysfunction; the remaining patients had acceptable postoperative courses.

## 4. Discussion

The choice between immediate and staged reconstruction was guided by the patient’s clinical status, infection control, and tissue condition. In patients without severe contamination or caustic injury, immediate reconstruction was considered safe. In others, a two-stage strategy allowed for stabilisation and healing prior to definitive reconstruction. OP is a potentially life-threatening condition with a reported mortality rate ranging from 10% to 25% when promptly diagnosed and treated [28]. This study analysed our experience with OP, focusing on surgical outcomes and the clinical variables influencing mortality, such as age, aetiology, location of perforation, oesophageal tissue condition, and extent of contamination.

Age over 75 years is a critical factor in the “Severity Score” for OP [15], guiding treatment decisions. In our series, 50% of deaths occurred in patients over 80 years old. The outcome is strongly associated with comorbidities.

In contrast to larger studies like that of Sdralis et al., which reported iatrogenic perforations as the most frequent cause [4,5], our series showed foreign body ingestion to be the leading diagnosis. This discrepancy may be due to the limited patient cohort and aligns with other retrospective studies, such as that of Deng et al. [29]. Mortality rates in our cohort did not vary by aetiology: caustic ingestion—two; foreign body ingestion—two; traumatic perforations and Boerhaave’s syndrome—two; iatrogenic perforations—no deaths reported.

The absence of deaths in iatrogenic cases, despite their high mortality in other studies [2,5,28], may reflect the early diagnosis and prompt treatment in our cohort. The timing of medical support plays a significant role.

Thoracic oesophagus perforation was the most common diagnosis (53.33%), consistent with the current literature [29,30,31]. However, cervical perforations showed a higher mortality rate than typically reported, possibly due to contamination severity extending to the mediastinum and pleural cavities, which impaired direct repair feasibility.

The overall mortality rate was 13.33%, in line with the numbers reported in the literature, and complications occurred in 66.66% of cases, confirming the disease’s severity [2,28,30]. Early diagnosis and treatment significantly influence outcomes. Primary oesophageal repair showed positive results, particularly within the first 24 h, though success also depended on the patient’s overall health [2,29].

While primary repair is typically reserved for early perforations (<24 h), some studies showed effectiveness even after 24 h [31,32]. However, delayed treatment increases risks of complications like mediastinitis, oesophagopleural fistulas, and sepsis. Buttressing sutured leaks remains critical given the limited improvement seen with alternative techniques [16,17,18,19].

Patients undergoing thoracoabdominal oesophagectomy with staged reconstruction had good outcomes. Orringer’s stomach tubulisation technique was used in two cases [33], while coloplasty was necessary in five patients, one of whom had a prior gastric resection [34]. Oesophageal resection has a mortality rate ranging from 13% to 66% [33], underscoring challenges with extensive oesophageal injuries, especially involving caustic ingestion [35] or spontaneous rupture. Two-stage reconstruction (6–12 months after primary surgery) was preferred, allowing for infection resolution and clinical improvement before surgery [1,9,30]. However, some studies support immediate reconstructive surgery with positive results under specific conditions [24].

Our findings reveal that while prompt treatment (<24 h) positively impacts outcomes, mortality is heavily influenced by age, comorbidities, and clinical scenario complexity, rather than by timing alone. These findings support other studies suggesting that delayed diagnosis and treatment significantly worsen prognosis [36,37].

## 5. Conclusions

This study emphasises that early diagnosis and tailored surgical strategies improve outcomes in OP. Primary repair remains the preferred approach in stable patients. However, in cases of extensive injury or late diagnosis, debridement or staged resection and reconstruction are valid options. The outcomes in selected cases of bipolar exclusion suggest that this controversial strategy may be acceptable in selected patients. The use of a muscle flap is a valid option to consider, utilising the surrounding tissues that can be most readily mobilised during direct repair of an oesophageal laceration. Patients’ comorbidities and clinical condition remain the key factors influencing prognosis. Even when diagnosis and treatment are timely, the impact of comorbidities on outcomes is substantial and cannot be overlooked.

OP remains a multifactorial clinical challenge. The rarity of cases and the limitations in randomising acute patients prevent any robust assessments of prognostic factors. Future research should adopt a multidimensional approach to study OP management rather than focusing solely on individual variables. Larger studies are needed to validate our findings and to provide stronger evidence for optimal treatment strategies.

## Figures and Tables

**Table 1:** Demographic data and clinical manifestations.

***Demographic Characteristics of Cohort***	
Age (years)	66 ± 17.75
Gender (male)	31
Smoking	13
Cardiovascular disease	6
Diabetes	2
Neurological and psychiatric disorders	10
Oncological diseases	2
***Frequent clinical symptoms onset***	***N° Patients***
Chest pain	26
Vomiting	12
Fever	10
Abdominal pain	8
Dysphagia	5
Subcutaneous emphysema	5
Cervical pain	3
Sialorrhea	3
Respiratory failure	3
Dyspnoea	3
Hemodynamic instability	1
Xerostomy	1
Dysphonia	1
Rhinolalia	1
Melaena	1

**Table 2:** Localisation of perforation, surgery performed, and time elapsed from event to admission.

	FB (n%)	Boerhaave S (n%)	Caustic Ing. (n%)	Traumatic (n%)	Iatrogenic (n%)
***Location***					
Cervical	10 (66.66%)			1 (50%)	2 (40%)
Distal	5 (33.33%)	11 (73.33%)	4 (50.00%)	1 (50%)	3 (60%)
Oesophagogastric		4 (26.66%)	4 (50.00%)		
***Surgery***					
Debridement, drainage	5 (33.33%)	3 (20%)		1 (50%)	1 (25%)
Oesophagotomy/Direct suture	10 (66.66%)	7 (46.66%)		1 (50%)	4 (80%)
Muscle/Tissue flap	6 (40%)	3 (20%)		1 (50%)	1 (20%)
Oesophagectomy		4 (26.66%)	4 (50%)		
Oesophagectomy and gastrectomy			3 (37.50%)		
Direct bipolar exclusion		1 (15.55%)			
Bipolar oesophageal diversion		2 (13.33%)			
Dietary jejunostomy	1 (6.66%)	6 (40%)	7 (87.50%)		1 (20%)
Dietary gastrostomy			1 (14.28%)		
***Reconstructive Surgery***					
Gastroplasty in same operative session		2 (13.33%)			
Two-stage retrosternal gastroplasty		2 (13.33%)			
Two-stage retrosternal coloplasty		2 (13.33%)	3 (37.50%)		
***Time of onset***					
<24 h	8 (53.33%)	7 (46.66%)	4 (50.00%)	2 (100%)	4 (80%)
>24–72 h	3 (20%)	3 (20%)	3 (37.50%)		1 (20%)
>72 h	4 (26.66%)	5 (33.33%)	1 (12.50%)		

**Table 3:** Post-operative complications and mortality rates based on surgery timing and flap location.

Post-Operative Complications	*N° Patients*	
Fever	17	
Fistula	7	
Respiratory failure	6	
Surgical wound infection	2	
Pleural and mediastinal effusion	2	
Oesophageal sub-stenosis	2	
Sepsis	1	
Mediastinitis	1	
DVT (deep vein thrombosis)	1	
Epistaxis	1	
Abdomen compartment syndrome	1	
***Flap group***	***Complications in N° of patients***	***p-value***
Cervical	(1/4) 25%	*0.303*
Thoracic	(4/7) 57.14%	
***Flap group***	***Mortality in N° of patients***	***p-value***
Cervical	(0/4) 0%	*0.428*
Thoracic	(1/7) 14.28%	
***Complication rates by time of onset***	***N° of patients***	***p-value***
<24 h	(15/25) 60%	*0.246*
>24 h	(14/20) 70%	
Mortality		
***Mortality rates by surgery timing***	***N° of patients***	***p-value***
<24	(6/25) 24%	***0.030***
>24	(0/20) 0%	

## Data Availability

The original contributions presented in this study are included in the article. Further inquiries can be directed to the corresponding author.

## Abbreviations

The following abbreviations are used in this manuscript:

OP	Oesophageal perforation
CT	Computed tomography
RE	Rigid oesophagoscopy
EGDS	Oesophagogastroduodenoscopy
ICU	Intensive care unit
FB	Foreign body
DTV	Deep vein thrombosis
